# Discovery of a novel small molecule as CD47/SIRPα and PD-1/PD-L1 dual inhibitor for cancer immunotherapy

**DOI:** 10.1186/s12964-024-01555-4

**Published:** 2024-03-11

**Authors:** Shengzhe Jin, Hongfei Wang, Yang Li, Jingwen Yang, Beibei Li, Peishang Shi, Xiangrui Zhang, Xiaowen Zhou, Xiuman Zhou, Xiaoshuang Niu, Menghan Wu, Yahong Wu, Wenjie Zhai, Yuanming Qi, Yanfeng Gao, Wenshan Zhao

**Affiliations:** 1 School of Life Sciences, Zhengzhou University, Zhengzhou, 450001 China; 2 Henan Key Laboratory of Immunology and Targeted Drugs, School of Medical Technology, Xinxiang Medical University, Xinxiang, Henan 453003 China; 3 School of Pharmaceutical Sciences (Shenzhen), Shenzhen Campus of Sun Yat-Sen University, Shenzhen, 518107 China; 4 International Joint Laboratory for Protein and Peptide Drugs of Henan Province, Zhengzhou University, Zhengzhou, 450001 China

**Keywords:** Tumor microenvironment, Immune checkpoint blockade, PD-1/PD-L1, CD47/SIRPα, Radiotherapy

## Abstract

**Background:**

Targeting the tumor microenvironment (TME) has emerged as a promising strategy in cancer treatment, particularly through the utilization of immune checkpoint blockade (ICB) agents such as PD-1/PD-L1 inhibitors. Despite partial success, the presence of tumor-associated macrophages (TAMs) contributes to an immunosuppressive TME that fosters tumor progression, and diminishes the therapeutic efficacy of ICB. Blockade of the CD47/SIRPα pathway has proven to be an effective intervention, that restores macrophage phagocytosis and yields substantial antitumor effects, especially when combined with PD-1/PD-L1 blockade. Therefore, the identification of small molecules capable of simultaneously blocking CD47/SIRPα and PD-1/PD-L1 interactions has remained imperative.

**Methods:**

SMC18, a small molecule with the capacity of targeting both SIRPα and PD-L1 was obtained using MST. The efficiency of SMC18 in interrupting CD47/SIRPα and PD-1/PD-L1 interactions was tested by the blocking assay. The function of SMC18 in enhancing the activity of macrophages and T cells was tested using phagocytosis assay and co-culture assay. The antitumor effects and mechanisms of SMC18 were investigated in the MC38-bearing mouse model.

**Results:**

SMC18, a small molecule that dual-targets both SIRPα and PD-L1 protein, was identified. SMC18 effectively blocked CD47/SIRPα interaction, thereby restoring macrophage phagocytosis, and disrupted PD-1/PD-L1 interactions, thus activating Jurkat cells, as evidenced by increased secretion of IL-2. SMC18 demonstrated substantial inhibition of MC38 tumor growths through promoting the infiltration of CD8^+^ T and M1-type macrophages into tumor sites, while also priming the function of CD8^+^ T cells and macrophages. Moreover, SMC18 in combination with radiotherapy (RT) further improved the therapeutic efficacy.

**Conclusion:**

Our findings suggested that the small molecule compound SMC18, which dual-targets the CD47/SIRPα and PD-1/PD-L1 pathways, could be a candidate for promoting macrophage- and T-cell-mediated phagocytosis and immune responses in cancer immunotherapy.

**Supplementary Information:**

The online version contains supplementary material available at 10.1186/s12964-024-01555-4.

## Introduction

The tumor microenvironment (TME) represents a complex and heterogeneous microecosystem comprising immune and non-immune stromal cells, providing tumor cells with sustained growth stimulus signals and nutritional support [1–3]. Targeting the TME is an effective strategy to inhibit tumor growth, particularly immune checkpoint blockade (ICB) by PD-1/PD-L1 inhibitors [4–6]. Unfortunately, only a minority of cancer patients benefits from ICB, partly due to the infiltration of immune cell subsets with inhibitory properties in the TME, such as Tregs, myeloid-derived suppressor cells (MDSCs) and tumor-associated macrophages (TAMs) [7, 8]. Of these subsets, TAMs represent the most predominant population of innate immune cells in the TME, and have been implicated in promoting tumor progression, metastasis, and recurrence through various mechanisms [9]. The increase of TAMs in the TME is often accompanied by the upregulation of immune checkpoints, including programmed death ligand 1 (PD-L1) and CD47. Therefore, targeting TAMs emerges as a potential therapeutic strategy for inhibiting tumor growth and improving treatment outcomes [10].

CD47, an immune checkpoint protein overexpressed on various cancer cells, binds to Signal Regulatory Protein alpha (SIRPα) on macrophages to deliver "don't eat me" signal that prevents phagocytosis of cancer cells [11]. Blocking the CD47/SIRPα pathway has been shown to activate macrophages and inhibit tumor growth [12]. In preclinical tumor-bearing mouse models, simultaneous blockade of CD47/SIRPα and PD-1/PD-L1 further inhibits tumor growth [13, 14]. Recent studies have highlighted the potential of dual-targeting CD47/SIRPα and PD-1/PD-L1 in inhibiting tumor growth [15]. Bispecific antibody drugs, fusion proteins and chimeric peptides with dual blocking effects on CD47/SIRPα and PD-1/PD-L1 interactions have demonstrated promising outcomes in preclinical and clinical studies, such as the bispecific antibody IBI322 [16], the fusion protein IAB [17] and peptide drug Pal-DMPOP [18]. However, CD47 is widely expressed in a variety of cells, particularly erythrocytes [19]. Targeting CD47 with antibody drugs may lead to "off-target " effects and elicit significant hematotoxicity [20]. Therefore, the development of small molecule compounds with reduced side effects and excellent drug efficacy is necessary for cancer treatment. Small molecule compounds, with the absence of Fc segment, can avoid antibody-dependent cell-mediated cytotoxicity (ADCC) side effects. Additionally, targeting SIRPα, which exhibits limited expression on the surface of myeloid cells such as macrophages and dendritic cells, can also avoid the "antigen sink" effects [21–23]. The identification of small molecules with dual-blocking effects on PD-1/PD-L1 and CD47/SIRPα interaction is crucial for cancer treatment.

Radiotherapy (RT) induces immunogenic death of tumor cells, promotes the exposure of pro-phagocytic signals and the release of tumor antigens, inducing macrophage phagocytosis and T-cell initiation [24–26]. Inhibitors targeting TAMs in combination with radiotherapy effectively enhance the antitumor immune response in clinical trials [27]. However, it is important to note that RT also upregulates PD-L1 expression in the TME, which may contribute to resistance and recurrence [28, 29]. Thus, exploring combinational strategies of CD47/SIRPα and PD-1/PD-L1 blockade with RT is necessary to achieve improved therapeutic efficiency.

In this study, we identified a novel small molecule compound, SMC18, capable of binding to both SIRPα and PD-L1, effectively blocking the CD47/SIRPα and PD-1/PD-L1 interactions. SMC18 effectively restored the phagocytic function of macrophages and enhanced the activity of T cells. Encouragingly, SMC18 also exhibited potent antitumor effects in vivo by promoting the infiltration and function of macrophages and CD8^+^ T cells at the tumor site, resulting in significant inhibition of tumor growth. Furthermore, our results suggested that SMC18 in combination with RT displayed even greater efficacy in suppressing tumor growth.

## Materials and methods

### Gene expression analysis

The single-cell sequencing data analysis of SIRPα and PD-1 in colon cancer patients and hormonal mice were analyzed using the website: http://crcleukocyte.cancer-pku.cn/. The correlation of SIRPα expression with patient survival, SIRPα and PD-L1 expression with CD14 and CD163 levels in colorectal cancer patients was analyzed using the data obtained from the Cancer Genome Atlas (TCGA) database. The differences of CD47 and PD-L1 expression in normal tissues and primary tumors of colorectal cancer (GSE37182) were analyzed using the data downloaded from the NIH Gene Public Database Expression Omnibus (GEO) (https://www.ncbi.nlm.nih.gov/geo/).

### Microscale thermophoresis (MST) binding assay

SIPRα-IgV-His (human: SIA-H5225, mouse: SIA-M52H4, ACRO Biosystems) and PD-L1-IgV-His (human: 10,084-H08H, mouse: 50,010-M08H, Sino Biological) proteins were labeled with Red-NHS647 dye (NT-MO-L001, NanoTemper) for subsequent experiments. SMC18 (C385-0664, ChemDiv) was serially diluted using PBST (from 0.0191 μM to 200 μM) and mixed with the labeled proteins. The samples were then aspirated using standard capillaries and assayed by MST instrument (Monolith NT.115, NanoTemper), and the results were analyzed using the analytical software Monolith NT.115 system to calculate the dissociation constant (K_D_) values.

### Blocking assays

The CHO-K1 cell line with stable expression of CD47 and PD-1 was constructed previously by our group [25, 30]. SMC18 was serially diluted using PBS 7.2 (containing 1% DMSO) and incubated with 100 ng of SIRPα-Fc protein (human: SIA-H5251, mouse: SIA-M5258, ACRO Biosystems) and 50 ng of PD-L1-Fc protein (human: 10,084-H02H, mouse: 50,010-M02H, Sino Biological) on ice for 30 min. The mixture was then added to the corresponding CHO-K1-CD47 or CHO-K1-PD-1 cells and further incubated on ice for 30 min. Afterward, PE-conjugated anti-human IgG (Fc) antibody (A18272, eBioscience) was added. The mean fluorescence intensity (MFI) of the cells was detected using flow cytometry (BD FACSCalibur) and the blocking efficiency was calculated.

### Molecular docking

The crystal structures of human SIRPα (PDB ID: 2UV3) and PD-L1 (PDB ID: 5C3T) were acquired from the Protein Data Bank database (PDB) (https://www.rcsb.org/). The 3D structure of SIRPα and PD-L1 were selected for docking followed by structure optimization with the software Molecular Operating Environment (MOE, Chemical Computing Group). Specifically, the binding pocket for small molecule inhibitors was selected with the Site Finder module. The compounds were prepared by adjusting protonation states and minimization prior to molecular docking. Molecular docking with induced-fit parameter was conducted within MOE.

### Phagocytosis assays

Bone marrow-derived macrophage cells (BMDMs) from C57BL/6N mice were isolated and induced with 20 ng/mL of mouse granulocyte–macrophage colony-stimulating factor (GM-CSF, 315–03, Peprotech) for 7 days, with regular medium changes and cytokine replenishment during cell induction. The cells were then harvested for subsequent experiments. Subsequently, the cells were treated with SMC18 at a concentration of 50 μΜ. The anti-CD47 antibody (clone miap301, 16–0471-81, Invitrogen) was employed as a positive control with 20 µg/mL. Phagocytosis was measured by the co-culture of macrophages with green fluorescent protein (GFP^+^) tumor cells (GFP^+^ MC38 and GFP^+^ B16-OVA cells) at a ratio of 1:4 in serum-free medium at 37 °C for 4 h. The cells were harvested and identified by flow cytometry using anti-F4/80 antibody (BM8, 17–4801-82, eBioscience). The phagocytosis rate was calculated as the percentage F4/80^+^GFP^+^ cells relative to the total number of total F4/80^+^ cells. For confocal microscopy detection, BMDMs were labelled with DiR dye (R-22070, Beyotime) at a final concentration of 200 μM, followed by a 30-min staining period. After washing with PBS three times, the cells were cultured with DMEM medium devoid of serum for 2 h. Subsequently, MC38-EGFP cells were seeded onto BMDMs at a density of 4 × 10^6^ cells per well. After co-culture for 1 h, the cells were washed and then fixed using a 4% paraformaldehyde solution for 30 min. The images were undertaken utilizing laser confocal microscopy (Carl Zeiss AG, LSM880).

### Co-culture assays

Jurkat cells were stimulated with 25 ng/mL of 12-myristate 13-acetate (PMA, 16,561–29-8, Sigma) and 1 μg/mL of phytohemag-glutinin (PHA, L8902, Sigma) or 1 μg/mL anti-human CD3 (OKT3, BioGems and 0.5 μg/mL anti-human CD28 (CD28.2, BioGems) for 24 h. Subsequently, they were co-cultured with CHO-K1-hPD-L1 cells at a ratio of 4:1 for 48 h. SMC18 (50 μΜ) and OPBP-1 peptide (100 μM) were added as experimental group and positive control group, respectively. Four hours before the end of the co-culture, protein transport inhibitor (555,029, BD Biosciences) was added. Jurkat cells were collected and stained with anti-human CD45 antibody (HI30, eBioscience) for 30 min. The cells were then fixed and stained with anti-human IL-2-APC (MQ1-17H12, eBioscience) for another 30 min. The fluorescence intensity was analyzed by flow cytometry (BD, FACSCelesta).

### MTT assay

MC38 and B16-OVA cells were seeded into 96-well plates at a density of 3,000 cells per well, respectively and allowed to adhere overnight. SMC18 was added to the cells and incubated for 24 h, 48 h and 72 h, respectively. Cell viability was measured by adding MTT (3-(4, 5-dimethyl-2-thiazolyl)-2,5-diphenyl-2-H-tetrazolium bromide, 298–93-1, Sigma) solution and incubating for 4 h. The cells were then lysed by shaking with DMSO. The absorbance values at 490 nm were measured using a multi-mode microplate reader (Molecular Devices, SpectraMax iD5).

### Tyrosine phosphorylation assay

Murine bone marrow-derived macrophages were subjected to induction using murine GM-CSF for 7 days. Subsequently, the induced macrophages (2 × 10^6^ cells/well) were co-cultured with MC38 cells (1 × 10^7^ cells/well). The co-cultures were treated with PBS (negative control), SMC18 (50 μM), or anti-mouse CD47 (clone miap301, 20 μg/mL, positive control) and incubated at 37℃ for 30 min. Following this, cells were washed and lysed. Finally, cell lysates were collected for immunoprecipitation and Western blotting.

Jurkat cells were stimulated with of PMA (25 ng/mL) and PHA (1 μg/mL) for 24 h. Stimulated Jurkat cells (2 × 10^6^ cells/well) were co-cultured with CHO-K1-PD-L1 cells (1 × 10^7^ cells/well). The co-cultures were treated with PBS, SMC18 (50 μM), or anti-human PD-1 (a17188b, Biolegend) and incubated at 37℃ for 30 min. After PBS washing, the cells were collected by centrifugation and lysed. Finally, cell lysates were collected for immunoprecipitation and Western blotting.

### Western blotting

In brief, the protein samples were separated by SDS-PAGE using a 4% stacking gel and an 8 ~ 10% main gel and transferred to a polyvinylidene difluoride (PVDF) membrane. The membrane was blocked with 5% milk at room temperature for 1–2 h and then incubated with anti-SIRPα (dilution 1:1000, AB8120, Abcam), anti-PD-1 (dilution 1:200, ab52587, Abcam) or anti-Phosphotyrosine (dilution 1:1000, 05–321, Millipore) dissolved in primary antibody dilution buffer overnight at 4 °C. Then the membrane was washed and incubated with appropriate HRP-coupled goat anti-rabbit IgG (D110058, Sangon Biotech) and HRP-coupled goat anti-mouse IgG (D110087, Sangon Biotech) for 1–2 h at room temperature. Finally, the membranes were exposed using an enhanced chemiluminescence (ECL) detection kit (P1030, Applygen). Results were analyzed by ImageJ software (NIH, Bethesda, MD, United States).

### Tumor models and treatments

The animal studies were conducted in accordance with the guidelines and approved by the Ethics Committee of Zhengzhou University (ZZUIRB 2021–36). Six weeks old female mice were purchased from the Vital River Laboratory (Beijing, China). Mouse MC38 colorectal cancer cells (1 × 10^6^) were harvested and subcutaneously (*s.c.)* injected into the right abdomen of the mice. When the tumor size reached 40 ~ 80 mm^3^, the mice were randomly assigned into three groups (*n* = 5 per group) to receive intraperitoneal injections daily of SMC18 (2 mg/kg), SMC18 (6 mg/kg) or normal saline for 14 days. At day 23, mice were euthanized for ex vivo analysis.

For the combined radiotherapy treatment model, mice were randomly grouped into four groups when the tumor volume reached approximately 40 ~ 60 mm^3^. Mice were treated with a single dose of 20 Gy of local tumor irradiation (Precision X-Ray, XRad320) and daily intraperitoneal injections of SMC18 (2 mg/kg) or equivalent amount of saline for 14 days. Tumor volume was measured every other day using the formula V = 1/2 × a × b × c, where a, b, and c are the tumor's length, width, and height, respectively.

### Ex vivo assays

At the end of treatment, the tumor-bearing mice were euthanized, and the tumor tissues were removed, minced and enzymatically digested with DNAase I (DN25, Sigma) and collagenase IV (17,104–019, Invitrogen) at 37 °C for 40 min. The digested tumor suspensions were filtered by nylon filter. Immune cell infiltration in the tumor tissue was analyzed by flow cytometry. For the detection of CD8^+^ T cells at the tumor site, single-cell suspensions were stained with anti-mouse CD45-FITC (30-F11, eBioscience), anti-mouse CD3-PerCP-eFluor710 (17A2, eBioscience), anti-mouse CD8α-PE (53–6.7, eBioscience) or isotype control. To detect macrophage infiltration at tumor sites, single cell suspensions were stained with anti-mouse CD45-FITC (30-F11, eBioscience), anti-mouse CD11b-eFluor450 (M1/70, eBioscience), anti-mouse F4/80-PerCP-Cyanine5.5 (BM8, eBioscience), anti-mouse CD11c-APC (M418, eBioscience), anti-mouse CD206-PE (MR6F3, eBioscience) or isotype control.

To assess cytokine secretion from tumor sites and immune organs, spleens, draining lymph nodes and tumors from mice were dissected and ground into single cell suspensions, followed by filtration and incubation with protease transport inhibitors, PMA (20 ng/mL, 16561–29-8, Sigma) and ionomycin (1 µM, I0634, Sigma) for 4 h in a 37 °C incubator. The single cell suspensions of draining lymph nodes and spleens were stained with anti-mouse CD3-PerCP-eFluor710 (17A2, eBioscience), anti-mouse CD8α-PE (53–6.7, eBioscience), anti-mouse IFN-γ-APC (XMG1.2, eBioscience) or isotype control. Tumor cells were stained with anti-mouse CD45-FITC (30-F11, eBioscience), anti-mouse CD3-PerCP-eFluor710 (17A2, eBioscience), anti-mouse CD8α-PE (53–6.7, eBioscience), anti-mouse IFN-γ-APC (XMG1.2, eBioscience) or isotype control. Finally, the samples were detected by flow cytometry.

### Mouse blood indicators, H&E staining and immunohistochemical analysis

Peripheral blood samples were collected from mice that received intravenous injections of SMC18 (2 or 6 mg/kg) for 2 weeks. The samples were analyzed for whole blood hematological test to detect the indicators of anemia and liver injury. For H&E staining analysis, tumors and the major organs (heart, liver, spleen, lung and kidney) were harvested from tumor-bearing mice. The tissues were then fixed with 4.0% paraformaldehyde for paraffin embedding and processed by H&E staining for histological evaluation. Immunostaining of tumor tissue sections was conducted utilizing anti-mouse CD8α andtibody (53–6.7, eBioscience).

### Cell lines and cell culture

The mouse colorectal cancer cell line MC38, the Chinese hamster ovary cell line CHO-K1 and the human T-lymphocyte line Jurkat were kindly presented by Prof. Sheng-Dian Wang (Institute of Biophysics, Chinese Academy of Sciences). CHO-K1 cells stably overexpressing human or murine PD-1 or CD47 (named CHO-K1-hCD47, CHO-K1-mCD47, CHO-K1-hPD-1, CHO-K1-mPD-1, respectively) were established by lentiviral transfection. CHO-K1 cells, Jurkat cells and melanoma cell line B16-OVA with EGFP (GFP^+^ B16-OVA) were cultured in Roswell Park Memorial Institute (RPMI) 1640 medium (11875093, Gibco). MC38 and melanoma cell line MC38 with EGFP (GFP^+^ MC38) were cultured in Dulbecco's Modified Eagle's Medium (DMEM) (11965092, Gibco). All cell culture media were supplemented with 10% fetal bovine serum (FBS, 04–001-1ACS, BI), 100 U/mL penicillin (P8420, Solarbio) and 100 μg/mL streptomycin (P1400, Solarbio). The cells were cultured at 37 °C and 5% CO_2_ in humidified incubator.

### Statistical analysis

Statistical analysis was performed with paired or unpaired two-tailed Student’s *t*-test. Data were represented as means ± S.E.M, with significant differences defined as *P < 0.05, **P < 0.01, and ***P < *0.001*.

## Results

### High expression of SIRPα and PD-L1 in colorectal cancer tissues

**Fig. 1:**
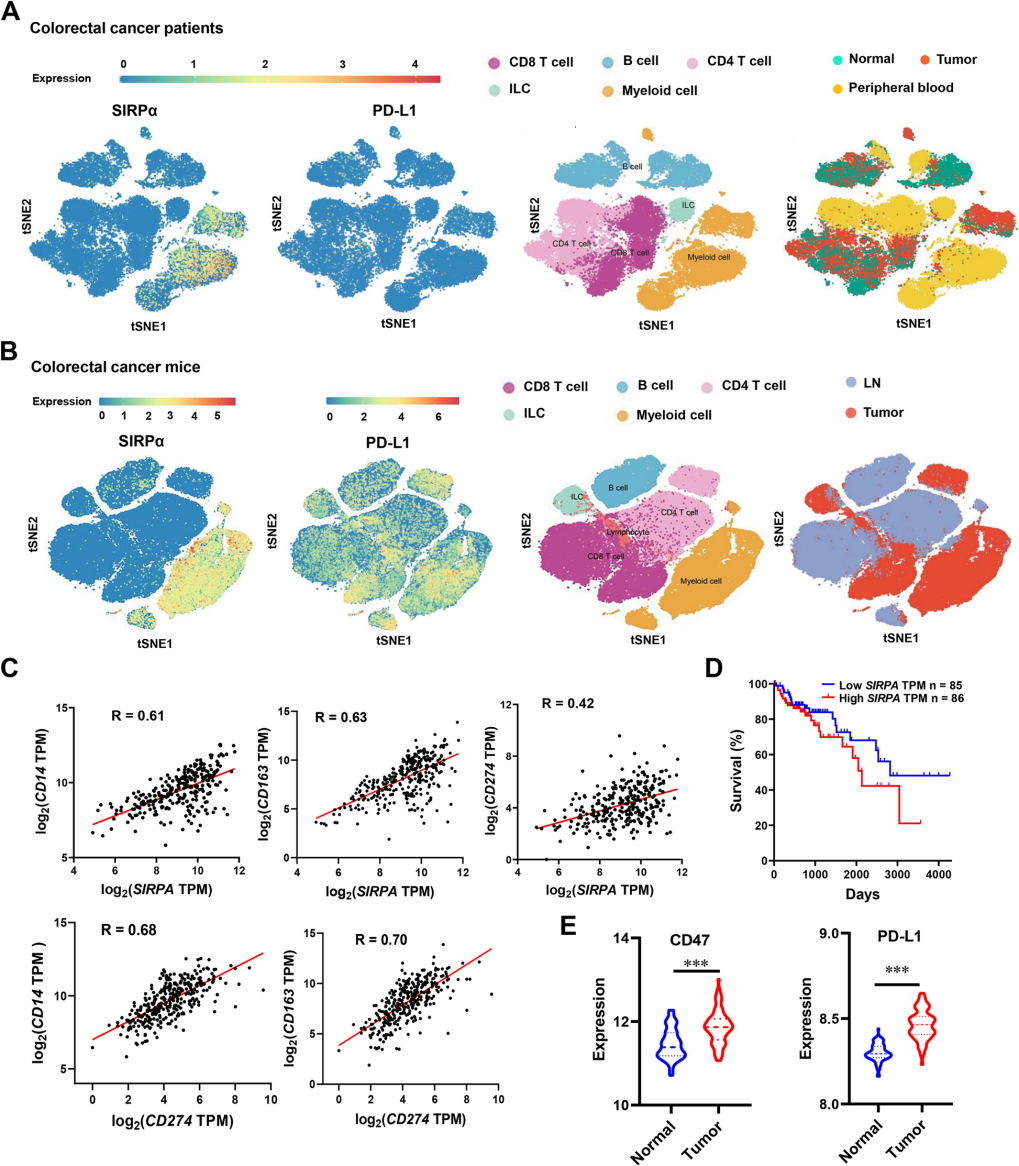
Expression analysis of SIRPα and PD-L1 in colorectal cancer tissues. **A** Expression of SIRPα and PD-L1 in Normal (N), PBMC (P), and tumor (T) tissues of patients with colorectal cancer (http://crcleukocyte.cancer-pku.cn/). **B** Expression of SIRPα and PD-L1 in normal and tumor tissues of mice with colorectal cancer (http://crcleukocyte.cancer-pku.cn/). **C** The correlation of SIRPα and PD-L1 with CD14 and CD163, as well as PD-L1 and SIRPα in colorectal cancer patients analyzed using the TCGA database. **D** The relationship between SIRPα expression and patient survival in the TCGA database for colorectal cancer. **E** Alterations in CD47 and PD-L1 in normal human samples and colorectal cancer patients from the GEO database (GSE37182). Statistical significance was determined using the unpaired Student's* t*-test (***P < 0.001)

To comprehensively investigate the expression profiles of SIRPα and PD-L1 in tumors, we conducted a thorough analysis. Firstly, we analyzed the single-cell RNA sequencing data from colorectal patients (Fig. 1A) and colon cancer mouse models (Fig. 1B). The results showed that SIRPα was primarily expressed in myeloid cells, whereas PD-L1 was expressed in CD4^+^ T cells, CD8^+^ T cells and myeloid cells (Fig. 1A ~ B, Fig. S1A ~ B). Moreover, SIRPα was found to be highly expressed in tumor tissues compared to normal tissues (Fig. S1B). In addition, we found that both SIRPα and PD-L1 were positively correlated with the expression of leukocyte differentiation antigen CD14 and macrophage marker CD163 from the TCGA database, suggesting that both proteins are associated with macrophages. Notably, the expression of SIRPα was also positively correlated with that of PD-L1 (Fig. 1C), and high expression of SIRPα was also associated with poor prognosis (Fig. 1D). This suggested that it is feasible to target both proteins simultaneously. Furthermore, analysis of RNA-seq data from the GEO database showed that CD47 and PD-L1 expression were significantly higher in colorectal cancer patients than in the general population (Fig. 1E). Taken together, our results suggested that CD47 and PD-L1 were highly expressed in tumor tissues and might play important roles in the progression of colorectal cancer.

### SMC18 exhibits strong affinity for SIRPα and PD-L1 proteins and effectively blocks ligand binding

**Fig. 2:**
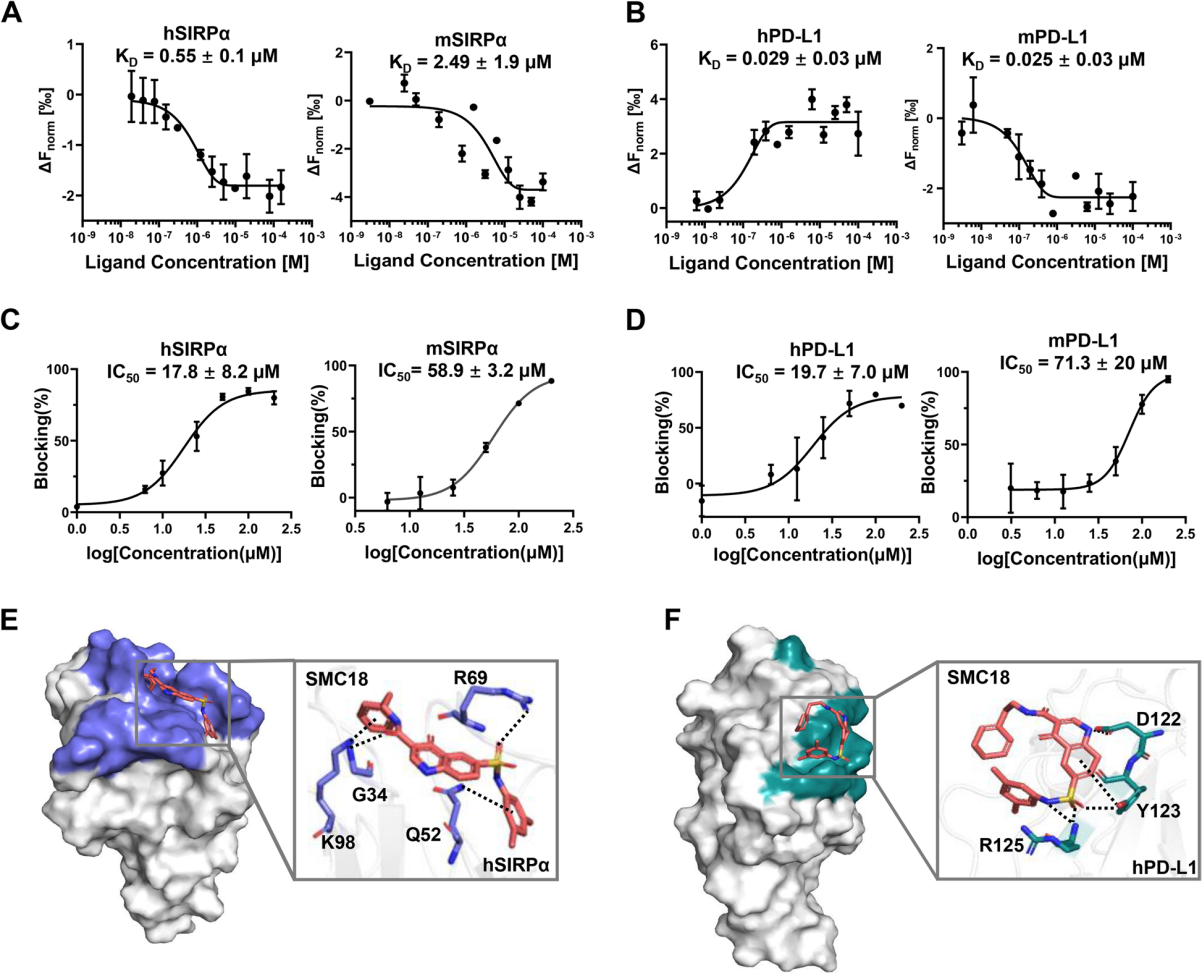
SMC18 binds SIRPα and PD-L1 to block CD47/SIRPα and PD-1/PD-L1 interaction. **A**, **B** The affinity of SMC18 with SIRPα and PDL1 proteins was assessed using MST. Dose curves were generated and presented. **C**, **D** Flow cytometry was employed to evaluate the ability of the SMC18 small molecule to block CD47/SIRPα and PD-1/PD-L1 interactions. The fluorescence intensity of CD47/PD-L1-Fc protein binding to cells with stable expression of SIRPα/PD-1 was measured at different concentrations of the small molecule, and concentration curves were constructed. **E**, **F** Interactions between SMC18 (orange-red) and amino acids derived from human SIRPα **E** and PD-L1 **F** were displayed. The specific amino acid residues involved in the CD47/SIRPα (blue-gray) and PD-1/PD-L1 (cyan-blue) interactions were indicated

Using the binding check module of microscale thermophoresis (MST), we conducted a comprehensive screening of our solid drug library and identified SMC18 as a small molecule with the ability to simultaneously bind to SIRPα and PD-L1. The dissociation constant (K_D_) values of SMC18 for human SIRPα (hSIRPα) and human PD-L1 (hPD-L1) were determined to be 0.55 ± 0.1 µM and 0.029 ± 0.03 µM, respectively, while the K_D_ values for murine SIRPα (mSIRPα) and murine PD-L1 (mPD-L1) were 2.49 ± 1.9 µM and 0.025 ± 0.03 µM, respectively (Fig. 2A ~ B). Next, a cell-based multi-concentration blocking assay was conducted to evaluate the ability of SMC18 to interfere with the interaction between CD47/SIRPα and PD-1/PD-L1. The results demonstrated that SMC18 effectively blocked CD47/SIRPα interaction in a concentration-dependent manner, with IC_50_ values of 17.8 ± 8.2 µM for human CD47/SIRPα and 58.9 ± 3.2 µM for murine CD47/SIRPα, respectively. Similarly, SMC18 dose-dependently disrupted the ligation between PD-1 and PD-L1, with IC_50_ values of 19.7 ± 7.0 µM for human and 71.3 ± 20 µM for murine interactions (Fig. 2C ~ D). The findings suggested that SMC18 has the ability to target both SIRPα and PD-L1, effectively interrupting their interactions with cognate ligands. Furthermore, we also assessed the inhibitory effects of SMC18 on other immune checkpoint pathways. The results demonstrated that SMC18 did not impede the hTIGIT/hPVR, hLAG-3/hFGL-1, hCD112R/hCD112, and hTIM-3/hGal-9 pathways (Fig. S2). This, to a certain extent, substantiated the specificity of SMC18 for blocking CD47/SIRPα and PD-1/PD-L1 interactions.

To gain insights into the mechanism underlying SMC18's dual-targeting effects on SIRPα and PD-L1, we conducted molecular docking analysis by software molecular operating environment (MOE). Our results indicated that SMC18 could interfere with CD47/SIRPα ligation by interacting with key residues G34, Q52, R69, and K98 of hSIRPα protein situated on the binding surface of CD47/SIRPα (Fig. 2E). Additionally, SMC18 bound to PD-L1 by interacting with residues D122, Y123, and R125 positioned at the binding interface of PD-1/PD-L1 complex (Fig. 2F). Collectively, our findings suggested that SMC18 exhibited the potential to effectively bind to both SIRPα and PD-L1, consequently disrupting CD47/SIRPα and PD-1/PD-L1 interactions.

### SMC18 effectively enhances the macrophages-mediated phagocytosis of tumor cells

**Fig. 3:**
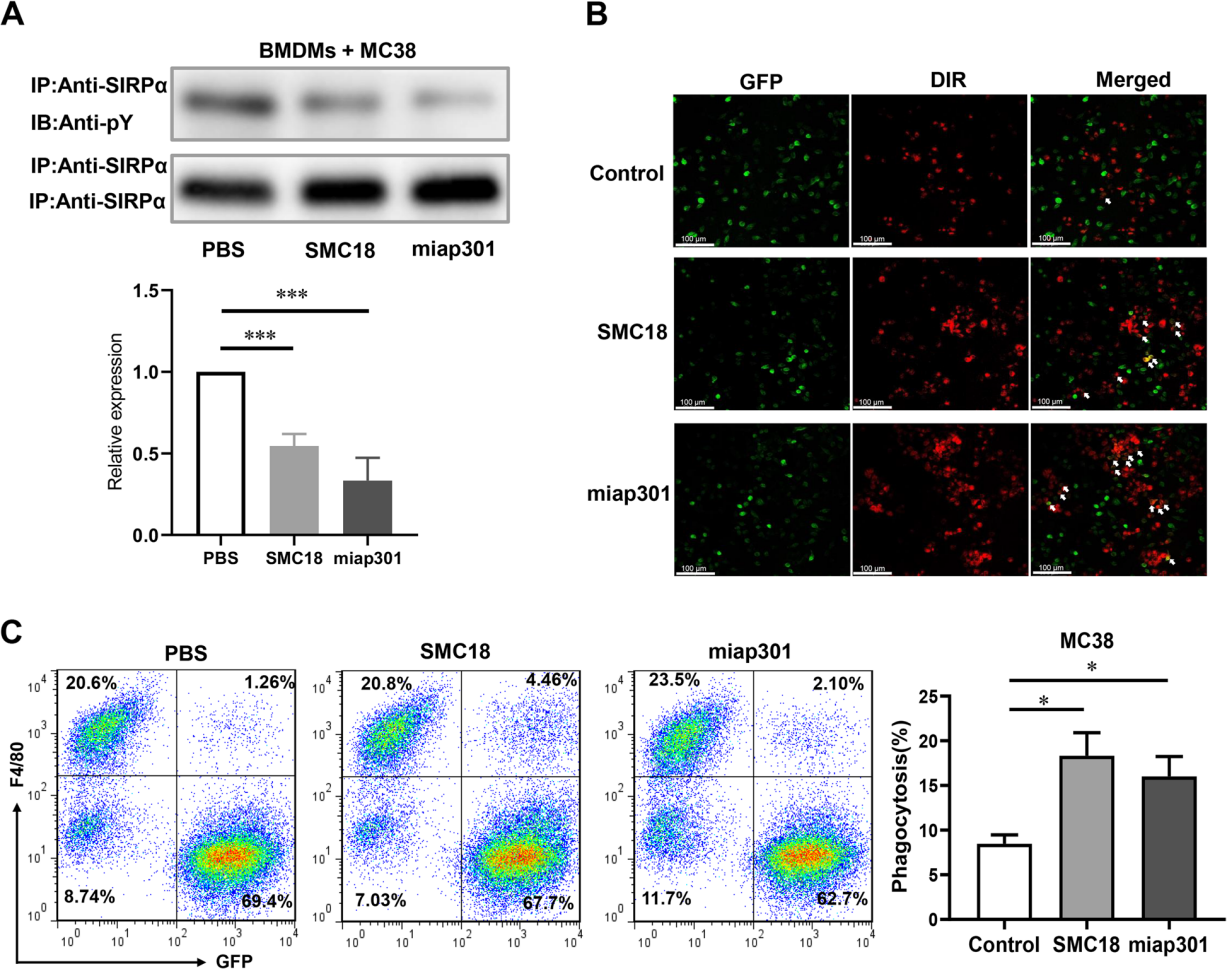
SMC18 increases macrophage phagocytosis of MC38 cells in vitro. **A** Representative Western blotting of SIRPα phosphorylation in the co-culture system of bone marrow-derived macrophages (BMDMs) with MC38 cells treated with PBS, SMC18 (50 μM), and anti-mouse CD47 (clone miap301, 20 µg/mL). Miap301 was set as a positive control and PBS as a negative control. IP (immunoprecipitation), IB (immunoblotting), and anti-pY (anti-phosphotyrosine). **B** Confocal images of bone marrow-derived macrophages from mice were labeled with DiR red dye and cultured with EGFP-labeled MC38 tumor cells. The cells were incubated with PBS, SMC18(50 μM) or miap301 (20 µg/mL). Representative images are shown. Arrows pointed to phagocytosed tumor cells. **C** Effects of SMC18 on phagocytosis of macrophages. Phagocytosis assays were conducted using EGFP-labeled tumor cells and mouse bone marrow-derived macrophages at a ratio of 1:4. SMC18 was used at a concentration of 50 μM. Co-cultured GFP^+^MC38 cells were assessed for the percentage of GFP^+^ macrophages using flow cytometry. The phagocytosis rate was calculated using the formula (GFP^+^ and F4/80^+^ cells)/F4/80^+^ cells. *n* = 3. The data are presented as means ± S.E.M. Statistical significance was determined using the unpaired Student's *t*-test (*P < 0.05, **P < 0.01, ***P < 0.001)

CD47, an immune checkpoint protein highly expressed on tumor cells, interacts with SIRPα on macrophages and induces phosphorylation of SIRPα to transmit a "don't eat me" signal, enabling tumor cells to evade immune surveillance [31]. To further validate the biological activity of SMC18, we confirmed that SMC18 was non-toxic to MC38 cells at a concentration of 100 µM, exhibiting only mild toxicity towards B16-OVA cells by MTT assays (Figure S3). We selected a relatively safe concentration of 50 µM for our subsequent experiments, as this concentration does not affect the viability of tumor cells. Initially, employing a co-culture system of bone marrow-derived macrophages (BMDMs) and MC38 cells, we measured the impact of SMC18 on the tyrosine phosphorylation of SIRPα. The results showed that SMC18 significantly suppressed the phosphorylation levels of SIRPα (Fig. 3A). CD47/SIRPα blockade can restore macrophage phagocytic activity. To confirm, we investigated the ability of SMC18 to enhance macrophage phagocytosis. Firstly, mouse colorectal cancer cell line MC38 stably expressing GFP were cultured with BMDMs stained with DiR in the presence of SMC18. The confocal results showed that SMC18 significantly enhanced macrophage phagocytosis of MC38 cells compared with the control group (Fig. 3B). Also, the flow cytometry results showed that SMC18 significantly increased the phagocytosis of MC38 (Fig. 3C) by BMDMs. Additionally, we co-cultured melanoma tumor cell line B16-OVA with BMDMs. The results showed that SMC18 significantly increased the phagocytosis of B16-OVA cells by BMDMs (Fig. S4). Collectively, SMC18 effectively restored macrophage phagocytic activity, suggesting the potential of SMC18 as a therapeutic agent for augmenting macrophage phagocytosis.

### SMC18 effectively restores the function of Jurkat cells in secreting IL-2

**Fig. 4:**
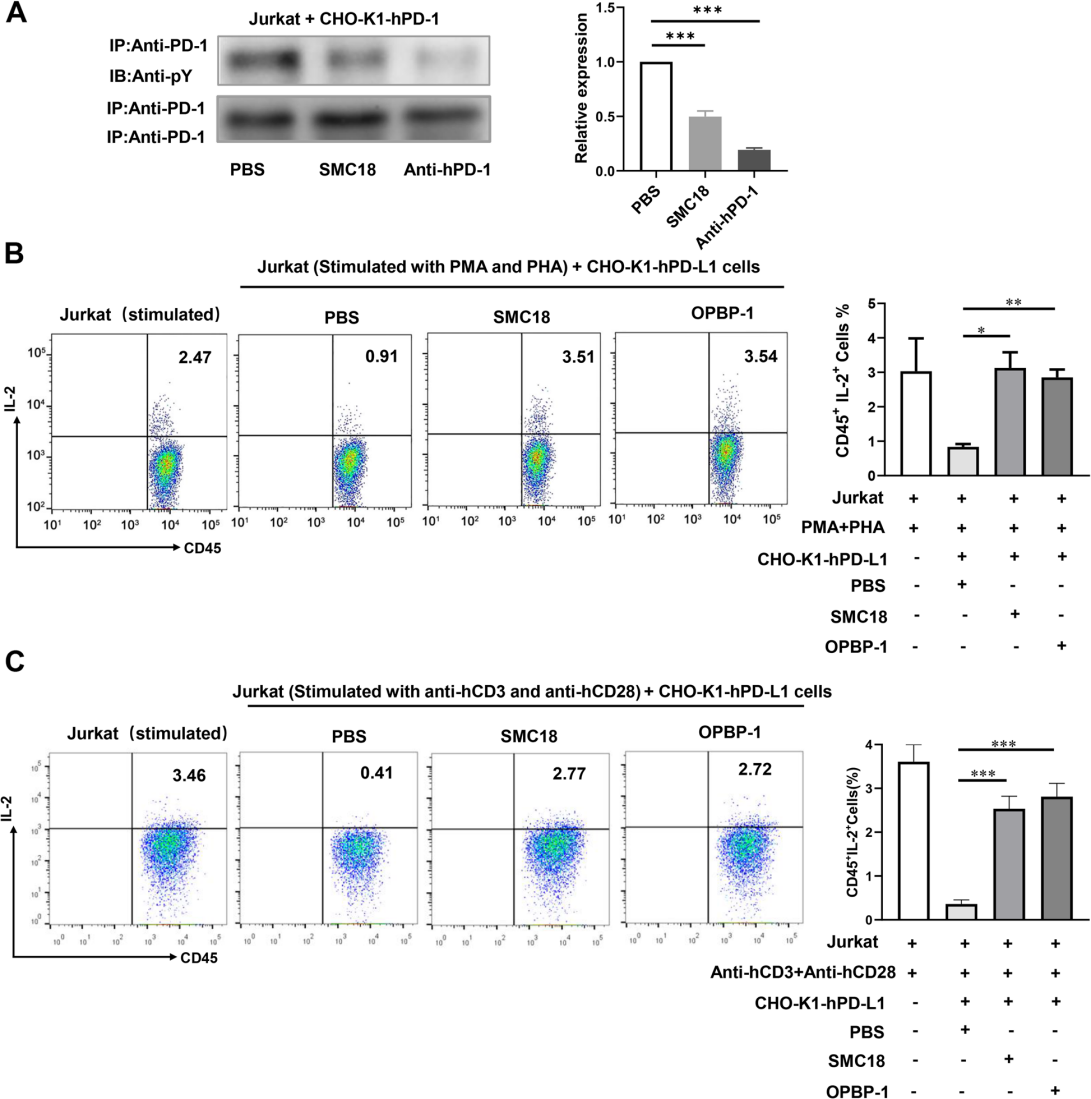
SMC18 effectively inhibits the phosphorylation of PD-1 and restores T cell function. **A** Representative Western blotting of PD-L1 phosphorylation in the co-culture system of Jurkat (stimulated with PMA and PHA) with CHO-K1-hPD-L1 cells treated with PBS, SMC18 (50 μM), and anti-hPD-1 antibody. IP (immunoprecipitation), IB (immunoblotting), and anti-pY (anti-phosphotyrosine). *n* = 3. **B** Representative flow cytometry and the pooled data of IL-2 secretion in co-culture experiments of Jurkat (stimulated with PMA and PHA) with CHO-K1-hPD-L1 cells. **C** Representative flow cytometry and the pooled data of IL-2 secretion in co-culture experiments of Jurkat (stimulated with anti-hCD3 and anti-hCD28) with CHO-K1-hPD-L1 cells. The data are presented as means ± S.E.M. Statistical significance was determined using the unpaired Student's *t*-test (*P < 0.05, **P < 0.01, ***P < 0.001)

It has been reported that the binding of PD-L1 possesses the capacity to induce tyrosine phosphorylation within the intracellular domain of PD-1, thereby propagating inhibitory signals that effectively curtail the functionality of T cells [32]. The blocking assay demonstrated the capacity of SMC18 to interfere with PD-1/PD-L1 interaction (Fig. 2D). In the co-culture system involving Jurkat and CHO-K1-hPD-L1 cells, we examined the impact of SMC18 on the tyrosine phosphorylation of PD-1. The results demonstrated that the introduction of SMC18 decreased the levels of phosphorylated PD-1 (Fig. 4A). To further evaluate whether SMC18 could restore T cell function by preventing PD-1/PD-L1 ligation, we co-cultured Jurkat cells (stimulated with PHA and PMA or anti-hCD3 and anti-hCD28) with CHO-K1-hPD-L1 cells in the presence of SMC18, and the IL-2 secretion by Jurkat cells was analyzed using flow cytometry. The PD-1/PD-L1 blocking peptide OPBP-1 was used as a positive control [30]. The results indicated that SMC18 effectively restored the ability of Jurkat cells to secrete IL-2 at a concentration of 50 μM, which was comparable to the activity of the OPBP-1 peptide (Fig. 4B and C).

### SMC18 significantly inhibits MC38 tumor growth and triggers antitumor immune response

**Fig. 5:**
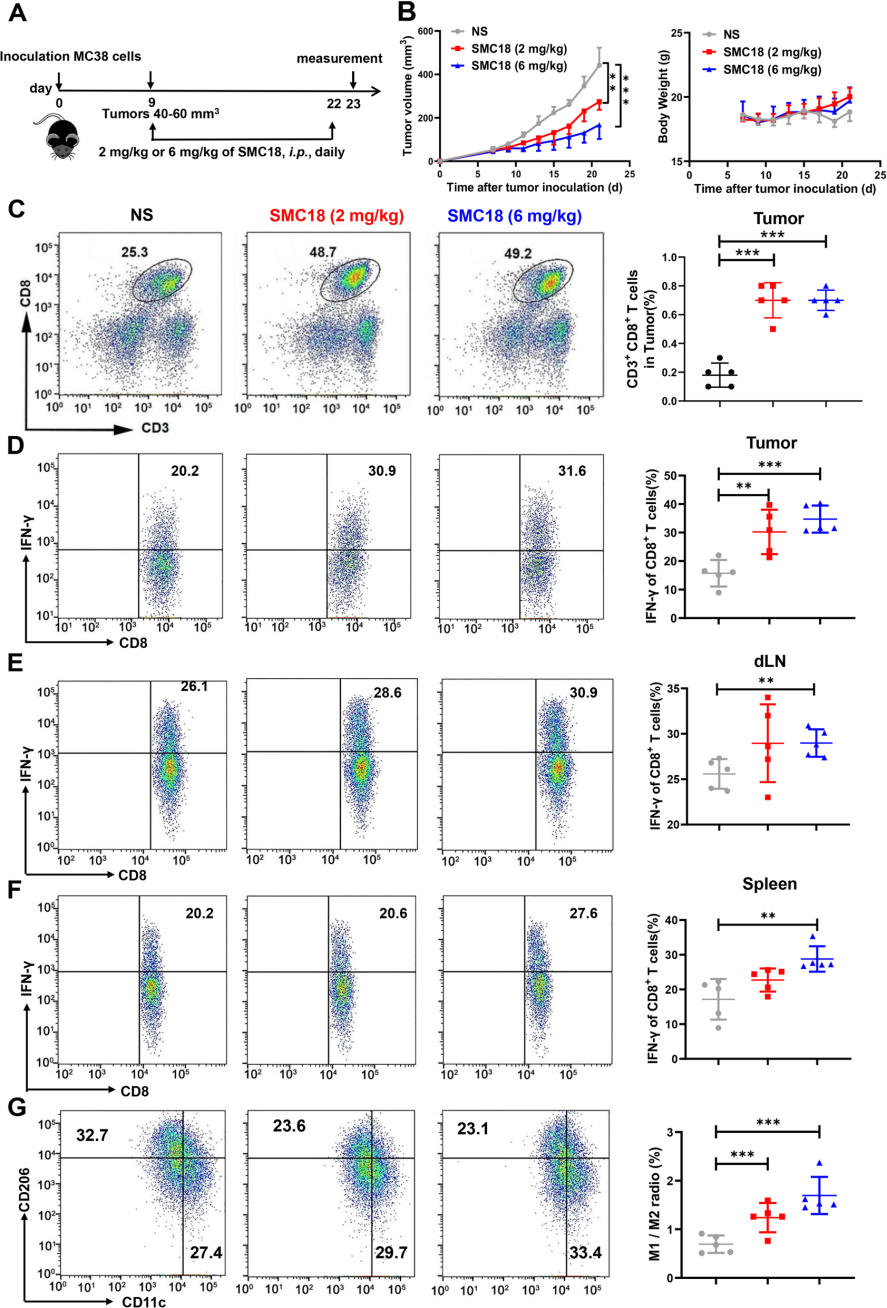
SMC18 effectively inhibits MC38 tumor growth and enhances immune cell infiltration into tumor site, promoting IFN-γ secretion. **A-B** Experiment scheme **A**, tumor volume **B** (left) and body weight **B** (right) in MC38 tumor-bearing mice treated with normal saline (NS), SMC18 (2 mg/kg) and SMC18 (6 mg/kg). **C** The proportion of CD8^+^ T cells in tumor sites of mice were measured by flow cytometry. **D**, **F** The proportion of CD8^+^ T cells secreting IFN-γ evaluated using flow cytometry. Tumor-infiltrating lymphocytes **D**, draining lymph nodes **E**, and spleen **F** were isolated from the mice and stimulated with 20 ng/mL PMA, and 1 μM ionomycin for 4 h. Following the incubation period, the cells were collected and fixed to disrupt the cell membrane, and the proportion of CD8^+^ T cells secreting IFN-γ was evaluated using flow cytometry. **G** Flow cytometry analysis of the proportion of M1-type macrophages ((CD45^+^F4/80^+^CD11b^+^CD11c^+^) and M2-type macrophages (CD45^+^F4/80^+^CD11b^+^CD206^+^) within the tumor sites, specifically in tumor-associated macrophages (CD45^+^F4/80^+^CD11b^+^). *n* = 5. The data are presented as means ± S.E.M. Statistical significance was determined using the unpaired Student's t-test (**P < 0.01, ***P < 0.001*)*

CD47/SIRPα and PD-1/PD-L1 have emerged as promising targets for cancer immunotherapy, and their combined blockade has shown superior antitumor effects in preclinical studies [33]. We established a MC38 tumor model, which is characterized by an abundance of intra-tumoral macrophages, to assess the antitumor potential of SMC18, a small molecule inhibitor targeting both CD47/SIRPα and PD-1/PD-L1 interactions. The treatment protocol is depicted in Fig. 5A. The results demonstrated that SMC18 effectively delayed the growth of MC38 tumors without affecting body weight of tumor-bearing mice (Fig. 5B).

To examine the effect of SMC18 on the antitumor immune response in mice, we analyzed the immune cell populations in the tumors, draining lymph nodes and spleens. The proportion of CD3^+^CD8^+^ T cells were analyzed according to the gate strategy (Fig. S5A). SMC18 treatment significantly increased the infiltration of CD8^+^ T cells (Fig. 5C, Fig. S5B ~ C) and the amount of CD8^+^ T cell secreting IFN-γ (Fig. 5D) in the tumor tissue. Furthermore, SMC18 treatment also increased the amount of CD8^+^ T secreting IFN-γ in the draining lymph nodes (Fig. 5E) and spleen (Fig. 5F), as well as the proportion of M1-type macrophages (Fig. 5G, Fig. S5D). Notably, the amount of IFN-γ secreting CD8^+^ T cells in the spleen was higher at a higher dose of SMC18 (Fig. 5F). These data collectively suggested that SMC18 effectively inhibited tumor growth by promoting the infiltration of M1-type macrophages and increasing the amount of CD8^+^ T cells secreting IFN-γ.

### SMC18 shows no hematotoxicity and hepatotoxicity

**Fig. 6:**
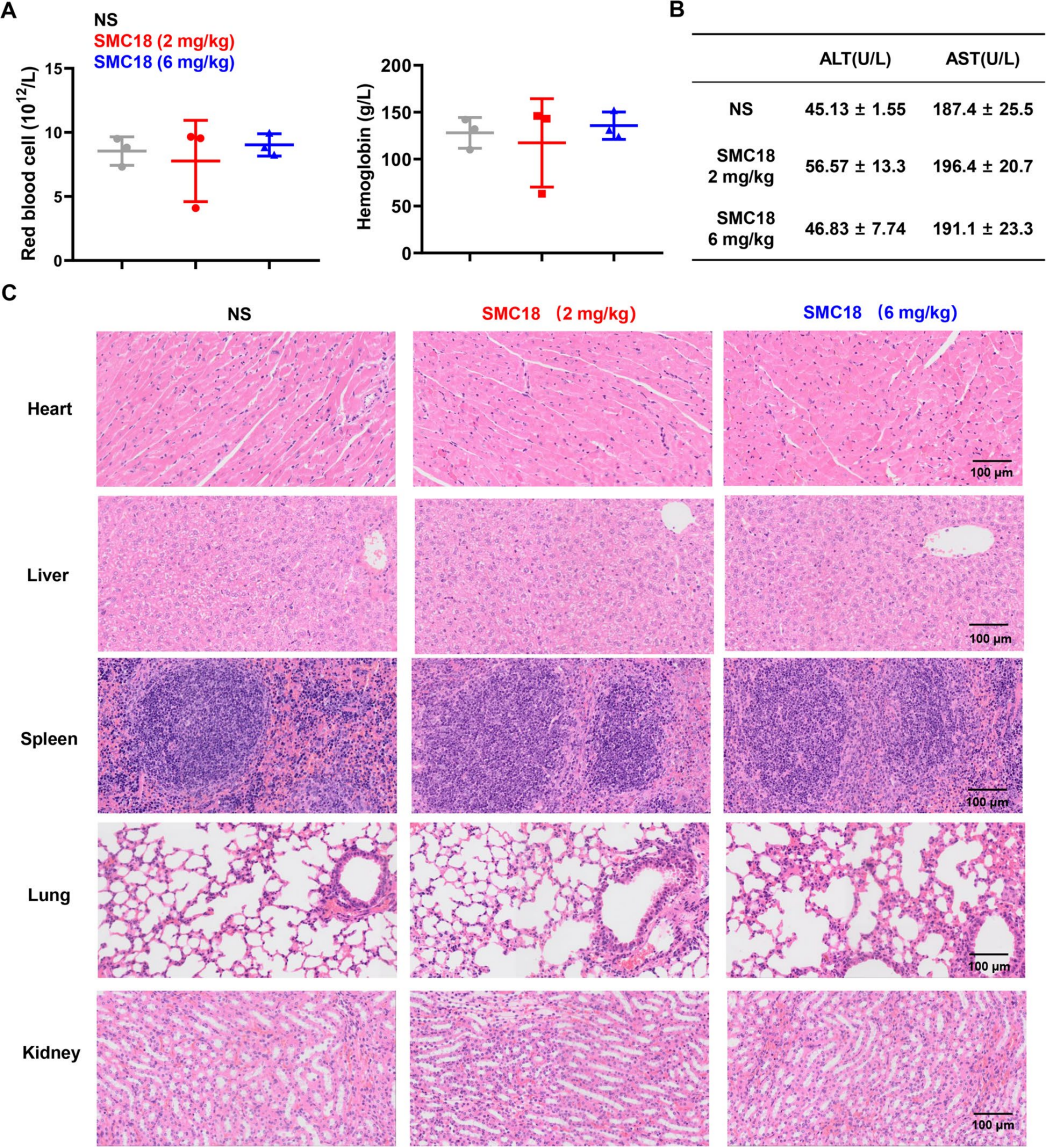
Histopathological analysis of hematotoxicity in mice. **A**, **B** C57BL/6 mice were subjected to daily intraperitoneal injections of the SMC18 or an equivalent saline solution for 14 days. Blood samples were collected and assessed for red blood cell (RBC) count **A** (left), hemoglobin levels **A** (right), as well as the levels of glutamic pyruvic transaminase (ALT) and glutamic oxaloacetic transaminase (AST) in mice **B**. **C** The major organs including the heart, liver, spleen, lung, and kidney were dissected from the mice, and tissue sections were prepared and stained with H&E prior to scanning. Representative images of the organ tissues were captured. (Scale bar: 100 μm)

The expression of CD47 on normal blood cells raises concerns regarding potential hematotoxicity when inhibiting the CD47/SIRPα interaction. To evaluate the safety of SMC18, we measured the levels of erythrocytes and hemoglobin in mice treated with SMC18. No significant differences between the SMC18 group and the control group were observed between the SMC18 group and the control group (Fig. 6A), indicating the absence of hematotoxic effects induced by SMC18 treatment. We also analyzed the content of alanine aminotransferase (ALT) and aspartate aminotransferase (AST) to evaluate the potential hepatotoxicity of SMC18 (Fig. 6B). Notably, no significant differences were observed (Fig. 6B), suggesting that SMC18 did not induce hepatotoxic effects. Additionally, H&E staining of major organs, including the heart, liver, spleen, lung, and kidney, showed no significant damage caused by SMC18 (Fig. 6C). These results collectively demonstrated the safety profile of SMC18, further supporting its potential as a promising therapeutic agent for cancer immunotherapy.

### SMC18 combined with radiotherapy further inhibits tumor growth

Radiotherapy is a well-established treatment modality in oncology, which are attributed to improved local tumor control and reduced disease dissemination. In addition to directly damaging tumor cells, RT also has an impact on the tumor microenvironment (TME), specifically activate immunosuppressive pathways of immune system cells. These effects culminate in the accumulation of radiation-resistant suppressor cells. Notably, local tumor irradiation has been shown to enhance the infiltration of MDSCs in tumor tissue. As precursors to macrophages, MDSCs differentiate into macrophages and promote the engulfment of tumor cells, thereby inhibiting tumor growth [34, 35]. Combining radiotherapy with CD47/SIRPα blockade can further enhance the phagocytosis of macrophages, leading to a significant reduction in tumor growth [36]. Moreover, radiotherapy induces upregulation of PD-L1, making it a potential candidate for combination therapy with SMC18, which targets the PD-1/PD-L1 pathway to elicit an antitumor response.

**Fig. 7:**
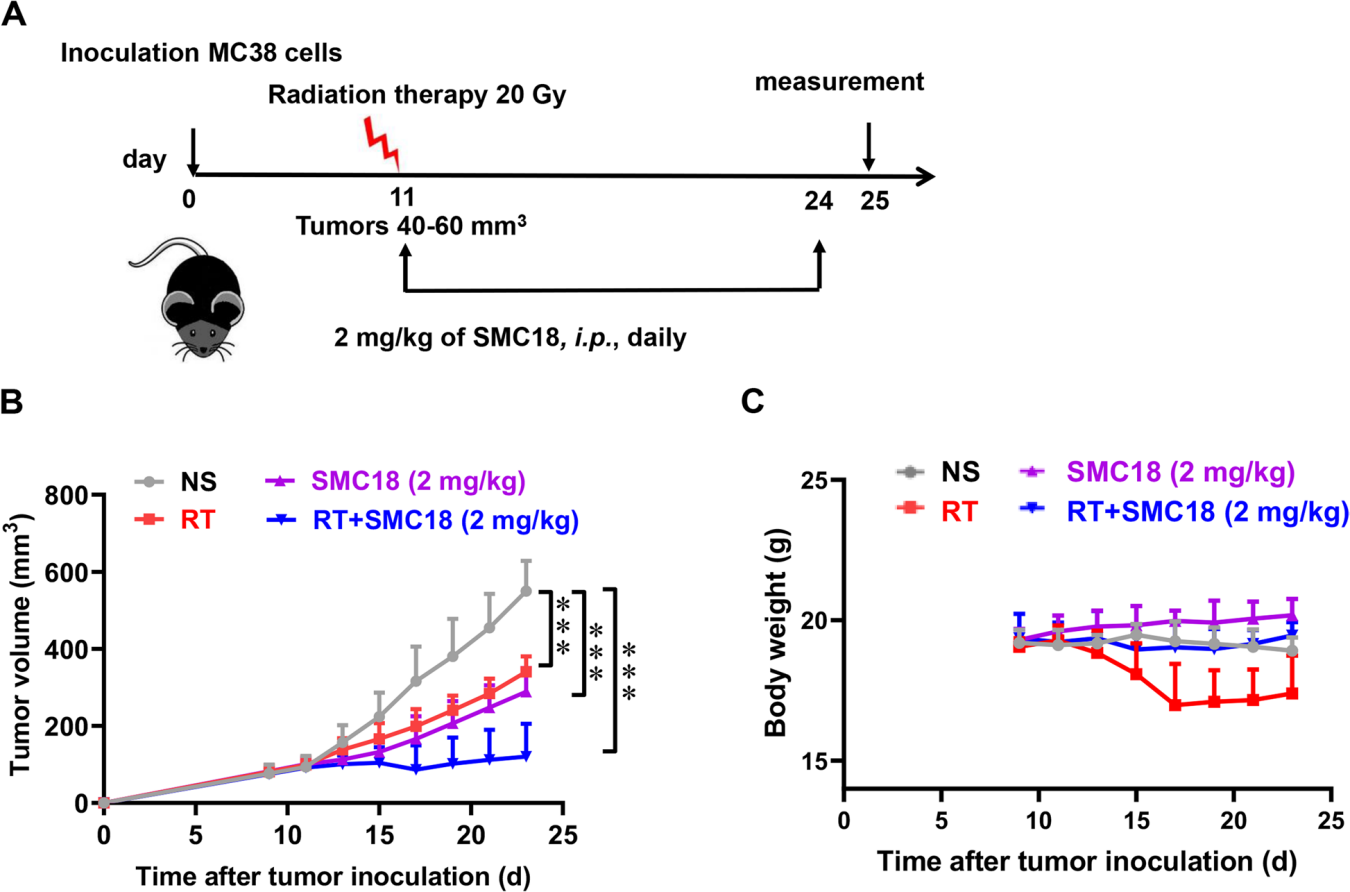
Synergistic inhibition of MC38 tumor growth by combining SMC18 with radiotherapy. **A-C** Experiment scheme **A**, tumor volume **B**, and body weight **C** in MC38 tumor-bearing mice. C57BL/6 mice were implanted with subcutaneous MC38 tumor cells on the right side. Tumors locally received one 20 Gy dose IR, and 2 mg/kg of SMC18 was daily *i.p.* administered for 14 days. Daily saline was set as a negative control. *n* = 5. The data are presented as means ± S.E.M. Statistical significance was determined using the unpaired Student's *t*-test (***P < 0.001*)*

To investigate the potential synergistic antitumor effects of SMC18 and radiotherapy, an in vivo experiment was conducted using mice bearing MC38 tumors. Once the tumor size reached 40–60 mm^3^, the mice received local radiotherapy at the tumor site followed by SMC18 treatment, as outlined in Fig. 7A. The results showed that the combination therapy significantly inhibited tumor growth (Fig. 7B), without affecting the body weight of the mice (Fig. 7C). Collectively, these findings highlighted the promising prospects of a dual blockade strategy targeting CD47/SIRPα and PD-1/PD-L1, in combination with radiotherapy, as an effective approach for achieving optimal tumor elimination.

## Discussion

Combined targeting of multiple immune checkpoints, particularly CD47/SIRPα and PD-1/PD-L1, has gained synergistic effects in cancer treatment [37, 38]. Analysis of single-cell sequencing data of human and mouse colorectal tumor tissues revealed elevated CD47 expression on myeloid cells and the expression of PD-L1 expressed on various immune cells (Fig. 1). SMC18, a small molecule with the ability to block CD47/SIRPα and PD-1/PD-L1 simultaneously, has the potential in augmenting innate and adaptive immunity against tumors.

The Fc fragment can bind to Fcγ receptors expressed on macrophages to trigger the activation of immune cells and elicit a synergistic tumoricidal effect through antibody-dependent cytophagy (ADCP) and ADCC [39]. However, SMC18 lacks the Fc segment and cannot activate the Fc-mediated effect. Nevertheless, phagocytosis experiments demonstrated the effectiveness of SMC18 in triggering phagocytosis (Fig. 3). Remarkably, CD47 is widely expressed on normal cells, especially on red blood cells and platelets. CD47 blockade may cause hematological toxicity. SMC18 bound to SIRPα with high affinity and showed no significant effects in erythrocyte and hemoglobin values (Fig. 6). Furthermore, the pathological analysis revealed no toxicity of SMC18 on major organs. In conclusion, SMC18 can effectively bind SIRPα to block CD47/SIRPα interaction and trigger phagocytosis, potentially serving as a promising approach for CD47/SIRPα blockade without side effects.

Bispecific antibodies (BsAb) can block two different targets simultaneously and have advantages over single-target inhibitors [40]. Currently, BsAb and peptide drugs predominantly target both CD47 and PD-1 or PD-L1, resulting in significant inhibition of tumor growth [15, 18]. However, drugs targeting SIRPα and PD-L1 simultaneously have not been reported yet. Our study revealed a positive correlation between SIRPα expression at the tumor site and PD-L1 in colorectal cancer patients (Fig. 1C), suggesting the rationale for simultaneously targeting SIRPα and PD-L1 in colorectal cancer treatment. While antibody drugs have some limitations, such as prolonged adverse effects due to the long half-life, small molecule compounds with low molecular weight and short metabolic half-life offer more manageable adverse effects. In addition, smaller molecules possess improved permeability, enabling easier entry into tumor cells [41].

RT has the potential to induce immunogenic cell death, thereby triggering an antitumor immune response. However, metastases frequently exhibit resistance to RT, resulting in high rates of recurrence following therapy. Moreover, RT can increase the expression of PD-L1 on cancer cells [29, 42], which can contribute to drug resistance. Additionally, RT can augment the infiltration of monocytes in the tumor microenvironment [25], which can subsequently differentiate into tumor-associated macrophages [34, 43]. Here, SMC18 could effectively block PD-1/PD-L1 interaction and CD47/SIRPα interaction to achieve a synergic effect in inhibiting tumor growth. Additionally, RT treatment can result in rapid weight loss due to the increased nutrient consumption by rapidly dividing tumor cells [44], thereby leading to decreased immunity and an increased risk of cancer recurrence and complications [45]. SMC18 had the potential to improve the effect of RT on body weight. However, it should be noted that small molecule inhibitors have certain drawbacks, such as a short half-life and the necessity for daily administration. Therefore, the modification of SMC18 to enhance its efficacy, in vivo half-life, and administration route requires further exploration.

In conclusion, our findings indicate that CD47 and PD-L1 are overexpressed across various tumor tissues, particularly in colorectal cancer. These results emphasize the considerable therapeutic potential of a combined blockade of CD47/SIRPα and PD-1/PD-L1 for the treatment of colorectal cancer. Within the framework of our study, we have identified a novel small molecule compound named SMC18, which efficiently targets critical residues within the CD47/SIRPα complex and PD-1/PD-L1 structure, simultaneously blocking the interactions between these proteins. Consequently, SMC18 significantly enhances macrophage phagocytosis of tumor cells, increases infiltration and restores the activity of CD8^+^ T cells in the tumor microenvironment, exhibits robust anti-tumor effects by inhibiting tumor growth with minimal side effects both in vitro and in vivo. Additionally, the combination therapy comprising SMC18 with radiotherapy exhibits even more pronounced therapeutic efficacy in suppressing tumor growth. This observation highlights the synergistic potential of this therapeutic approach. In summary, our research underscores the dual-targeting proficiency of the small-molecule compound SMC18 against both the CD47/SIRPα and PD-1/PD-L1 pathways, bridging innate and adaptive immunity and thus providing a promising candidate for cancer immunotherapy.

### Supplementary Information

**Supplementary Material 1.**

## Data Availability

Data and materials are available by contacting the corresponding author.

## Abbreviations

TME: Tumor microenvironment
TAMs: Tumor-associated macrophages
RT: Radiotherapy
ICB: Immune checkpoint blockade
MDSCs: Myeloid-derived suppressor cells
PD-L1: Programmed death ligand 1
ADCC: Antibody-dependent cell-mediated cytotoxicity
ADCP: Antibody-dependent cytophagy
MST: Microscale thermophoresis
MFI: Mean fluorescence intensity
BMDMs: Bone marrow-derived macrophages
GM-CSF: Granulocyte–macrophage colony-stimulating factor
GFP: Green fluorescent protein
PHA: Phytohemag-glutinin
PMA: Phorbol-12-myristate 13-acetate
RPMI: Roswell Park Memorial Institute
DMEM: Dulbecco's Modified Eagle's Medium
FBS: Fetal bovine serum
TCGA: The Cancer Genome Atlas
ALT: Alanine aminotransferase
AST: Aspartate aminotransferase
BsAb: Bispecific antibody
