# Supplementary material for: Discovery of a novel small molecule as CD47/SIRPα and PD-1/PD-L1 dual inhibitor for cancer immunotherapy
Source: Cell Commun Signal. 2024 Mar 11;22:173. doi: 10.1186/s12964-024-01555-4 (PMC10926604; doi:10.1186/s12964-024-01555-4)
Supplement: Supplementary file 1 — Supplementary Material 1. [file 12964_2024_1555_MOESM1_ESM.docx]

**Supplemental Data**


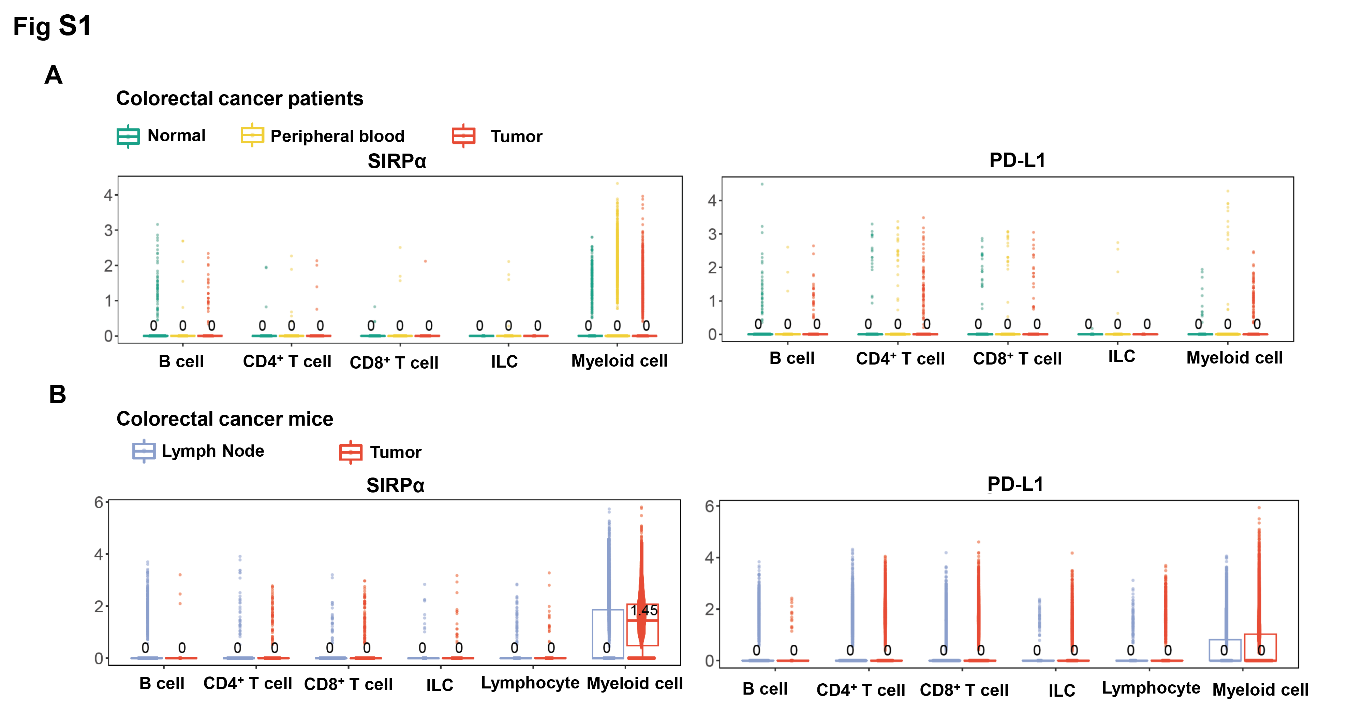


**Fig S1. SIRPα and PD-L1 expression in colorectal cancer samples.** **(A)** Expression of SIRPα (left) and PD-L1 (right) in normal tissues, peripheral blood, and tumors of colorectal cancer patients with different immune cells. **(B)** Different immune cell expressions of SIRPα (left) and PD-L1 (right) in lymph nodes and tumors of mice with colorectal cancer.


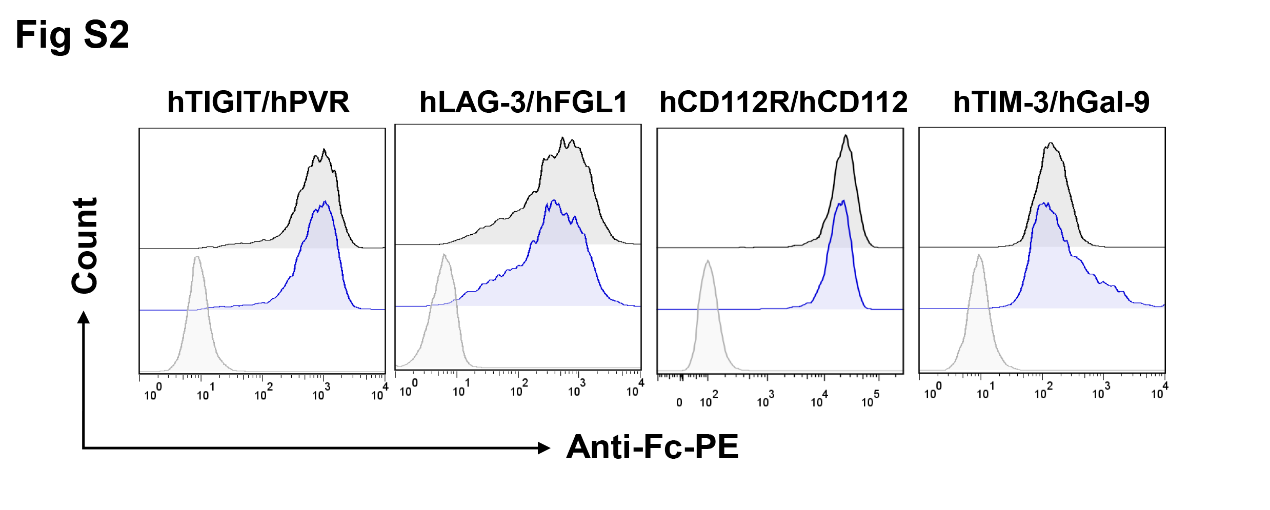


**Fig S2. Blockade of** **other immune checkpoint pathways by SMC18.** SMC18 exhibited no blocking effect on other immune checkpoint pathways including TIGIT/PVR, LAG-3/FGL1, CD112/CD112R, and TIM-3/Gal-9.


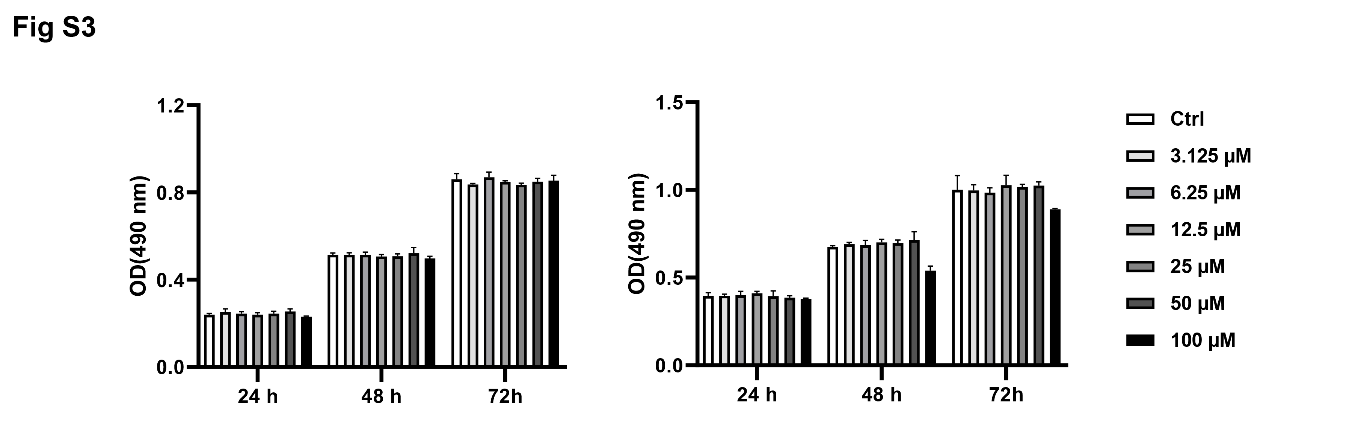


**Fig S3. The Effect of SMC18 on Tumor Cell Proliferation *in vitro*.** MC38 cells (3000 cells/well) and B16-OVA cells (3000 cells/well) were seeded in flat-bottomed 96-well plates and allowed to adhere overnight. Subsequently, the cells were starved for 8 hours in serum-free medium. SMC18 was diluted in complete culture medium and added to the wells, following which the cells were incubated for 24 h, 48 h, and 72 h. After the designated incubation periods, 20 μL of MTT solution was added to each well. Four hours later, the supernatant was aspirated, and 150 μL of DMSO was added to each well to dissolve the MTT formazan product. The plates were then shaken for 10 minutes, and absorbance was measured at 490 nm using a microplate reader.


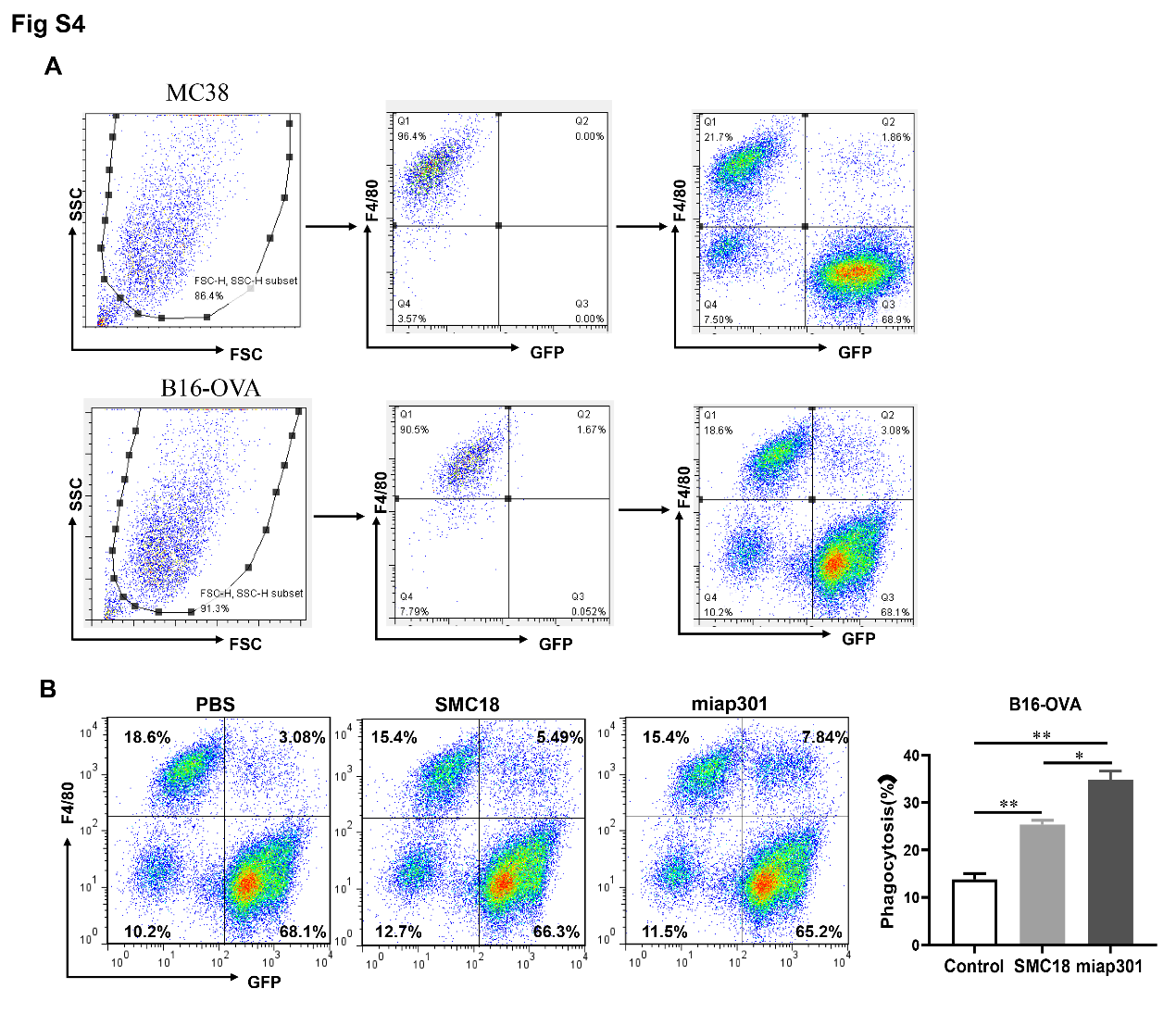


**Fig S4. Effects of SMC18 on phagocytosis of macrophages. (A)** Gate strategy for analyzing the proportion of GFP^+^F4/80^+^ macrophages cells. **(B)** SMC18 enhances the ability of macrophages to phagocytose B16-OVA cells.


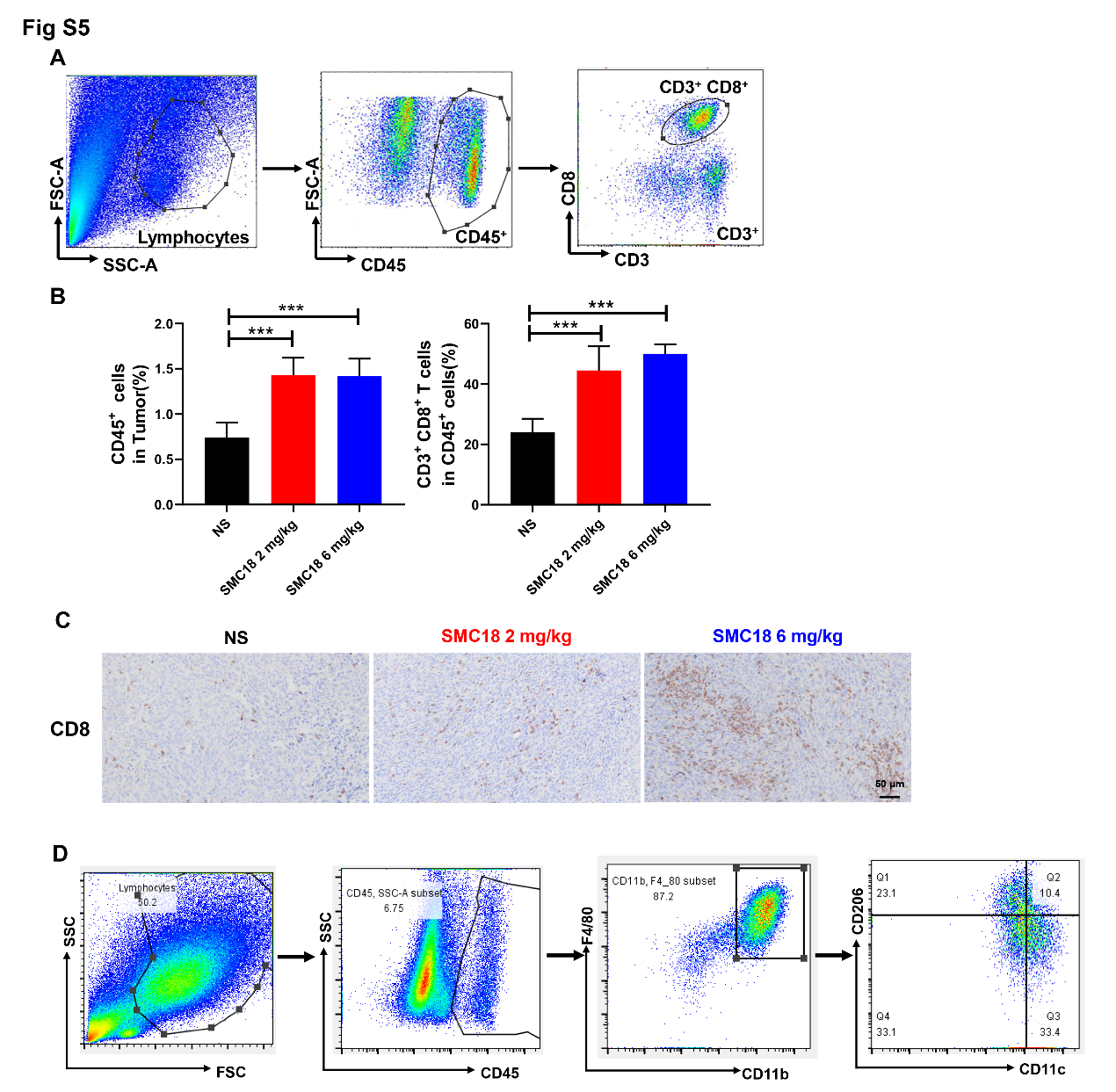


**Fig S5. SMC18 effectively potentiates immune cell infiltration into tumor sites. (A)** Gate strategy for analyzing the proportion of CD3^+^CD8^+^ T cells. **(B)** The proportion of CD45^+^ cells and CD8^+^ T cells in CD45^+^ cells in tumor were measured by flow cytometry. **(C)** Immunohistochemical analysis of CD8 expression in tumor tissue. Scale bar: 50 µm. **(D)** Gate strategy for analyzing the proportion of M1 macrophages cells (CD11b^+^ F4/80^+^ CD11c^+^) and M2 macrophages cells (CD11b^+^ F4/80^+^ CD206^+^).
